# A serious-gamification blueprint towards a normalized attention

**DOI:** 10.1186/s40708-021-00127-3

**Published:** 2021-04-15

**Authors:** Saad Alqithami

**Affiliations:** Department of Computer Science, Albaha University, Al Bahah, Saudi Arabia

**Keywords:** Agent-based modeling, Game design, ADHD, Cognitive behavioral therapy, Attention

## Abstract

Attention is an important commodity in the human skills set. It can be trained to overcome deficits in the short term which might be based on multiple cognitive complications to entail inability to keep focus and mined wondering. On the long term, however, it might be a symptom of chronic diseases that acquire attention to include the spectra of many mental health disorders, e.g., attention deficit hyperactivity disorder (ADHD). This paper, therefore, introduces a generic reference model that guides in the design of proper treatment method for patients in short of attention to engage in a game-based environment in order to enhance the behavior of their current state of attention which may hopefully lead to a better focus. When considering the volatility of traditional cognitive behavioral therapies (CBTs), the model reflects and analyzes evolving serious games design directed for the treatment of ADHD. It serves as an instrument that spawn over a specific treatment design since it introduces essential components that depicts essential units of traditional CBT when they are modularly combined. The components will be introduced and the processes of the reference model will be elaborated as a roadmap for the formation and the operation of augmented reality treatment games.

## Introduction

Reality, in a simple sense, reflects an actual existence processed into the brain through sensory inputs of the body. The stimuli we feel, see, hear, smell or even taste can shape a structure of our current reality in the brain. Hacking into the brain allows us to alter such structured realities through the manipulation of such sensory data [1]. Music, for instance, plays a big role in mood enhancements when triggering specific neurons at different regions of the brain. Here, immersive technologies that amalgamate the physical world with a digital/simulated reality comes into place, e.g., the virtual/augmented reality [2]. It is a computer-based simulated environment that allows the immersion into a real-life settings with the creation of simulated sensory surrounding world. Virtual Reality (VR) replaces a player environment with a more enforced/structured ones. Different virtuality levels can isolate the player from the real environment at one level or keep on continual engagements at another. Augmentation refers to the reality where virtual and real existence occur at the same time. Augmented Reality (AR) allows a player to engage in real-world activities while virtual inputs can be introduced throughout. The advantages that virtual/augmented reality instantly add helps for controlling the environment we live in since it allows us to alter the brain connectivities for better trajectories.

Cognitive Behavioral Therapy (CBT) or even Ramification Focused CBT are of many psychotherapy treatments for multiple psychological disorders. CBT, from a narrative point of view, challenge patients in repetitive motion to overcome unhelpful cognitive distortions observed in patients with attention dysfunctions, e.g., heterogeneous developmental disorder such as Attention Deficit Disorder (ADD) and Attention Deficit Hyperactivity Disorder (ADHD) [3, 4]. ADHD is a heterogeneous neuro-developmental disorder observed through three dimensions: impulsivity, hyperactivity and inattention leading to unsettling and long-lasting diagnostic of inadequate learning and adaptation. According to the evidence of anatomy and physiology, the cognitive process of human brain depends on the interaction and communication between both brain regions. In recent years, the development of neuroimaging technology provides an effective means to explore the functional or structural interaction between brain regions which is helpful to understand the pathological basis of neurological diseases. Brain network, therefore, reflects an innovative perspective to identify biomarkers to help in the automation of diagnosis for brain diseases.

### Attention and the brain

Hacking into the brain through stimulating different regions with the employment of simulated AR scenarios requires the understanding of human attention. Attention is a mental muscle that can be strengthened by exercising the brain. Thus, focus exercise is a natural method to strengthen the connectivity in the intentional circuitry of the brain with the utilization of economized attention through habituation or orientation at will. Circuitry of attention falls in between the prefrontal area, where a person concisely pay attention to what matters now and to make decisions and learn, and the emotional centers in the midbrain particularly the amygdala, a radar for danger that triggers proper strategies or strong emotions. Oscillators in the brain cells govern how a person respond to someone else and physical objects leading to full-mutual attention and non-verbal synchrony. Selective attention might be challenged with sensory and emotional distractors; however, disruptive attention makes it hard to build a cumulative mental model and to master any subject [5]. E.g., change in stress hormones like cortisol and adrenaline may contribute to low engagement level. Mind-wondering is sometimes valuable specially when a creative act makes a connection between remote elements in a new way.

The onslaught of digital world into our personnel universe has a side effect on childhood with constant distractions that lose their ability to comprehend. Brain is the last organ of the human body to become anatomically mature and during that development, the principle of neuroplasticity is important where repeated experiences shape the brain. E.g., when a person pay full attention and ignore distractions, the connectivity for that circuitry grows. Such an external growth is needed for children to develop their brain well. The capacity of the mental muscle of attention circuitry also calms stormy emotions; ability to manage emotion is inextricably linked with ability to pay attention. Socioemotional learning is a way for children to learn by themselves when they utilize the emotional intelligence component for self-awareness, empathy and handling relationships. Moreover, three views of focus are elementary for attention: (a) awareness of the current state of mind to make decisions utilizing ancient thoughts in base-brain and does not require connection to the part of thinking through words. It has rich connectivity to the gastrointestinal tract to the gut, i.e., semantic markers and reading body languages; (b) tune-in to others through cognitive, emotional and empathetic concerns; and (c) strategic thinking and formulation through exploitation and exploration.

Attention is observed to be with a direct correlation with emotion, where systematic stress can direct and support attention [6, 7]. Stress as a physiological response or a defensive mechanism was captured by [8] in the model of general adaptation syndrome in three stages of alarm, resistance and exhaustion [9]. While stress can be observed as a stimulus to trigger responsive actions for a better adaptation and learning [10], Richard Lazarus [11] developed the transactional theory of stress and coping as product to explain stress as more of a dynamic process between a patient and the complex environment. We benefit from this theory in the gamification design environment, where patients are directed to be aware of the introduced obstacles and proceed to overcome them to adapt to the ever-changing environment in their surroundings. To this end, we came to the realization that emotions direct attention and one way to trigger emotional states is through visual stimulus and the engagement with a challenging task.

### Non-cooperative serious games

Serious games are a reflection of traditional way of entertainment gaming with the purpose of directed therapeutic, educational, explorational, etc. incentives in their designs and implementations [12–15]. Such game design has been widely examined in the literature and researchers are still in the progress of defining parameters that are useful to direct those games [16–18]. Games are modeled in the field of Artificial Intelligence as search problems. Thus, heuristics are utilized to evaluate those games.

**Fig. 1:**
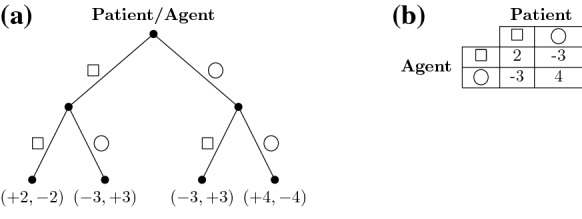
An abstract example of a patient engaging in a non-cooperative game with a software agent in both **a** an extensive-tree form and **b** a normal-matrix form of a 2-player and 2-strategy game

From a game theoretical perspective, games are considered cooperative or non-cooperative in nature. In cooperative games, players work together to achieve a common goal (e.g., a coalition formation); whereas on the contrary, in non-cooperative games, players challenge each other for greater good of having a higher payoff than another (e.g., stochastic games). The idea is working against an adversary to maximize ones own utility. The consideration of game theory unleashes the finding of an optimal policy when that policy can depend on the opponent policy and vice versa [19]. Therefore, the design of this paper considers noncooperative deterministic games when structuring the design of the augmented reality serious game. This will allow the patient for self-reliance and progress intentionally to succeed in the game, while cooperation exempts the player from continual engagements which contradicts the purpose of the game [20]. The player will engage in competition with an agent forming a two-player noncooperative game, which allows them to work against each other for the purpose to maximize their individual payoffs. Figure 1 present an abstract example of the two-player game described in details later in Sect. 2.

The example presented in Fig. 1 shows a two-payer game environment where one is the patient him-/herself, while another is the game itself. The software agent, as another player, reflects the intelligent system that operates the game. As both players engage in a zero-sum game, an agent will look into finding a dominant strategy that maximize its own payoff no matter what the patient choses. This will challenge the patient throughout the session, leading to an increase in the level of engagement. An outcome is observed once both players reach an equilibria state, where no player can benefit from switching to a different strategy assuming that the other player stays the same. In a simple sense of the game design, the shapes for the patient (i.e., $\Box$ and $\bigcirc$) are virtual solid objects collected from random locations at the AR environment. The shapes are transmitted to the destination area where shapes are moved into different halls that best fits them. The rule allows one shape to be transmitted at a time in a manner that best challenges the patient. As the idea here is to increase attention and focus for a longer time, patient will move from one location to another while holding a virtual object where time is calculated and distance is increased throughout the treatment session.

### Where we are in the literature

There is an ongoing rapid growth in the use and development of technology. A lot of attention has been focused on immersive technologies lately to surpass entertainments to cover more practical applications in military trainings, health assessments, medical rehabilitations, education, and many others [2, 21–24]. In light to this trend, we embark on the fact that children that are more likely to interact with technologies than most adults which pins out the possibility for better intervention tactics. Since the behavior and learning can essentially be enhanced through adjustments in the long-term memory, the authors in [25] evaluated the impact of immersive technologies on children’s memory (aged 6–7) in comparison with other types of storytelling and were able to find out that there is a better applicability to comprehend story content in certain situations when utilizing immersion. Such an advantage is perhaps due to fostering more realistic predefined scenarios for better visual engagements as examined by [26]. In fact, the paper in [26] did not report a significant improvement in the recall time and accuracy when shifting from directing the player to complete the session up to a complete subtraction of the visual stimuli that allow the player to be of a consistent and stable gazing pattern.

Immersive realities, i.e., VR and AR, blend the real world with fiction in a look-alike game scenario to provide more realistic experience for the user [27]. One application is the implementation of serious games that are semi-games designed at an early stage towards predefined objectives beyond entertainments [12]. Although they may satisfy the main purpose of gaming of motivation for a better engagement, they have a subsequent objective to be achieved as well such as special training and therapy [28, 29]. Gamification, as another application, presents the shift in the design of traditional games to a serious games [17]. Although it reflects the design of a game for special purpose, it can, however, be implemented at any stage of the design or implementation process to achieve the objectives of that shift, e.g., learning or applying new skills. We, therefore, ground this work on the stimulus–response theory to consider any experience as a formation of a gameplay [10]. The psychological theory reflects an agent’s behavior as a manifestation of stimulus and response interplay to create subsequent actions that reflect his/her behavior.

Emotion is the trigger of actions and reactions. Self-regulations of emotions are with deficit in patients with short attention, such as ADHD patients, since they lack inhibitory control due to their impulsivity. ADHD is a heterogeneous developmental disorder with currently not-well-defined etiologies that have led to unsystematic hyper-activeness, impulsiveness and distractions [30, 31]. As emotion and cognitive regulations support the treatment of ADHD, the utilization of immersive realities as an exposure therapy proposes potential benefits that have been extensively examined in the literature [32–37]. E.g., the DJINNI AR model serves as an exposure therapy to patients with social anxiety disorder [38]. The authors in [39] reported a significant increase in engagement as perceptual sensitivities and response biases have decreased in their VR rehabilitation experiment. The benefit of utilizing immersive technologies in learning and rehabilitation has also been extended to other mental disabilities to cover autism spectrum disorder [40], cerebral palsy [41], and spatial functioning in cerebral palsy [42] or even on a general note a postural control [43].

The research on software agents design and simulation has long-lasting history in serious game design as well as gamification and planning. The authors in [44, 45] propose a Java library for programming intelligent/autonomous test agents called APLIB to test games that are in nature inherent non-deterministic state space. Automated game testing was also discussed in [46], when the author deployed a user experience testing framework using belief, desire and intension test agents regulated through emotion. The importance of the simulation used in VR or AR is to predict the capability, limitation and performance of a system and, in result, will lead to a reduction in costs and hazards for experimentations compared with what is observed in traditional/hands-on methods [47, 48]. An enhancement of those immersive simulated realities can be achieved through agent-based modeling (ABM) and design. The use of ABM will produce a simulation with a high degree of fidelity [49]. In a study by [50], the authors implemented ABM in an augmented simulated environment using wearable computing devices and were able to develop a model that analyzes user activities and predict near-future possible needs. Another study, in [47], has proposed a new approach of introducing synthetic agents in motion at real time into a real-life video depending on a terrain database and graphical rendering.

In light of the rich literature, this paper will propose a blueprint that serves as generic reference model to test the validity of using an online immersive reality system that manifests behavior as an interplay between stimulation and response of actions as a CBT to feature qualities of intelligence, responsiveness, adaptiveness and precision. The previous works, presented in [51–53], stride implemented an augmented reality game environment using both Microsoft-HoloLens emulator^1^ and Unity^2^ to design a specialized test-bed for the system. The hypothesis tackled was that an increase in patient correct attention to choosing a predefined object contributes positively to their performance index which means they are following along with their treatment plan; the opposite should be true when they fail to achieve their assigned tasks. The paper went further to propose a measurement of the performance index based on multiple factors: correct response, impulsivity, inattention, engagement and errors throughout time slots. The plan in this paper is, however, to propose a more generic reference model that overcomes specialized applications for the treatment to be implemented efficiently. The rest of the paper will present an encapsulation of the formal modeling of major concepts and a highlight of the processes that combine proposed concepts together to construct this system from the ground-up in Sects. 2 and 3 summarizes and sheds the light on possible future directions that are still to be explored to understand and evaluate the proposed model.

## From conceptualization to modeling

We drive into forming the reference model through following baby-steps starting from defining the concept of our approach, to answering the questions of why we chose this method and to where we are heading. For the treatment to function properly, we build on the assumption that patients have already been diagnosed with the deficit in attention (i.e., ADHD) and have already established medical records with the hospital/clinic from where these experiments are conducted or to be supervised. We continue to omit the ethical guidelines to performing and experimenting with patients in this article, and assume that proper and ethically approved protocols have been followed in a hypothetical example.

### A generic design of the system

**Fig. 2:**
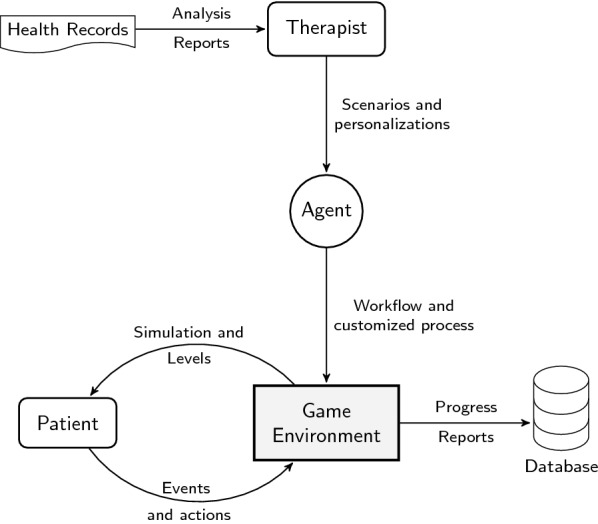
A system architecture that illustrates the main concept of the reference model

Besides from the two major elements of a treatment method to function that are therapists and patients, a proper methodology that fits a patient needs and requirements plays a major role in improving the progress of that treatment. Best suitable treatment application is not an easy task; therapists are able to set an approximate solution based on direct assessments and retrieved patient's medical records. In this architecture, we introduce an agent that is able to navigate within the treatment process to assist in the treatment plan as well as an alarm to report any improvements or lack of progress for better intervention tactics. The chance of a prompt intervention permits therapists to set policies and to navigate with a better suited therapeutic method for the patient towards complementing the main treatment processes. The software agent will set as a back layer between the treatment environment and the therapist to access scenarios and personalizations set by the therapist for a patient based on the health records and to keep with a continuous update within the game environment to feed recently updated workflow and to set up customized interactive processes for the treatment to function properly. The high-level design, shown in Fig. 2, represents the main components of the reference model and the therapist–patient relationships within the online game environment.

**Fig. 3:**
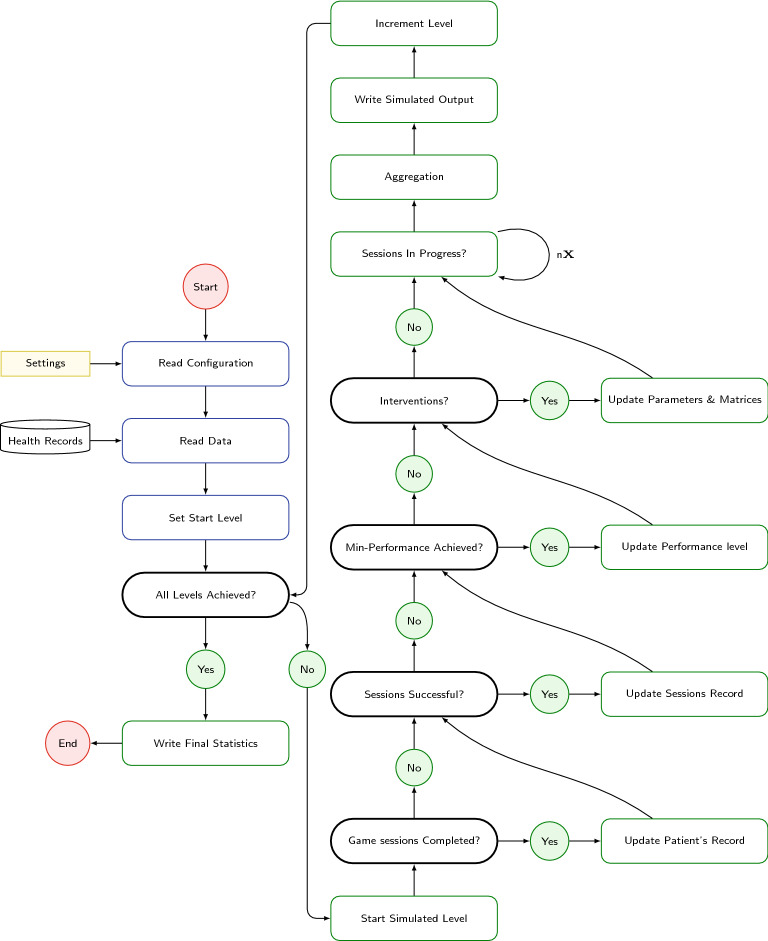
A diagram to illustrate the processes of a game activity life-cycle as a state in the reference model

Patients are able to engage with the game environment to perform a set of actions towards a completion of a given task that supports a planed treatment. While the game-based interactive environment stores data as a progress report directly to local/online servers for future retrieval and processing, it engages with an agent for on-time interference and customizations. The process within the propose conceptual architecture is presented in Fig. 3. The generic configurations are set based on the therapist recommendations to include many parameters like: the number of sessions, the starting game level, external effects, and conditions for transition from one level to the next one. As stated previously, the patients' health records are provided with the diagnostics from specialized therapists to include with their basic informations besides the preliminary assessments of their the current attention level and existing deficits. Other medical conditions are omitted in this model with the hope that therapist interventions overcome obstacles resulting from them if any or at least choosing the best fit treatment level and plan that works along their medical records. Patients play-to-win the game designed, and movement as well as actions are measured and analyzed to allow the therapist to monitor the treatment progress. After a patient starts a new game session, the model should trigger the interactive agent which performs two basic functions: controlling the game environment and monitoring the patient’s performance. The performance of the patient is characterized by many parameters, such as Gaming Time, Response Time, Standard Deviation of the response time, Score, Errors of Commissions, Errors of Omissions, and Response Sensitivity. After the player ends the game session, the agent will store the values of the parameters in the database. These values will be analyzed by the agent to derive some important performance indicators that decide on the next level to be assigned.

The therapist initiates the system by diagnosing the symptoms of ADHD and also setting up the treatment plan. The ADHD patient starts a new game session. The agent will request the game configuration from the database which is determined based on the treatment plan. The agent will collect the performance parameters while the game is in progress. After ending the game session, the agent will analyze the collected results and determine the values of performance indicators. The analysis results will be stored in the database in order to start the game level of the next session.

### Formal model

This section presents a set of formal definitions that conceptualize the reference model. In general, the environment is modeled as a game-based system where both patients and therapists are players. The game involves multiple patients who are under-supervision by their own therapists. Both players are not intervening with any other ones progress nor treatment plans while the session is in progress. There is no interaction between patients nor with their therapists in the game, and each session is played by one patient at a time or in parallel. In the game environment, a set of patients (i.e., $\hbox {Patient}_i \in \{Patient\}$) and a set of therapists (i.e., Therapist$_j \in \{Therapist\}$) are provided. The therapist assigns a level of complexity (i.e., $\hbox {Level}_s \in \{Level\}$) to the patient based on the diagnosis made in the treatment center.

The model can be abstractly observed as a tuple: $\langle$ Environment, $\hbox {Patient}_i$, $\hbox {Therapist}_j$, {Game Session}, {Object}, {Level} $\rangle$. Those six profiles play an important role in defining the model and are connected to one another to form the model. The following will present a detailed description for each one of them.

#### **Definition 1**

Environment, which is the functioning system for the treatment stage, is a tuple of $\langle$ {Object}, {Patient}, {Therapist}, {Level} $\rangle$, where:

- {Patient}: Each patient will have his/her own profile. The profile has to be complete for the patient to join the environment and start the game as stated in Definition 2.
- {Therapist}: Each therapist will have his/her own profile.
- {Object}: The set of virtual objects that a patient will interact with throughout the game. A detailed description of it is in Definition 6.
- {Level}: It defines the current mental state for the patient defined at an early stage by the therapist. Definition 5 gives more descriptive overview.

#### **Definition 2**

$\text{Patient }_i$ $\in${Patient} for whom this treatment is designed: $\langle$ $\hbox {ID}_i$, {Preference}$_i$, Performance-$\hbox {Index}_i$, $\hbox {Level}_s$, $\hbox {Location}_i$ $\rangle$

- $\text{ID}_i$: A short identification as a name or a referral number used to define a patient in treatment (i.e., used by a therapist to define a specific patient).
- ${\{\text{Preference}\}}_i$: The set of predefined preferences for a patient considering other psychological disorders that may affect current design and methodology of the treatment plan.
- $\text{Performance-Index}^i$: The current value of performance the patient has achieved throughout the game. It accumulated throughout multiple levels the patient has achieved within a session.
- $\text{Level}_s$: The level to where the patient has arrived in the treatment plan, which is $\in$ {Level} as presented in Definition 5. Since the set of the complexity levels that the patient has to go through is sorted and passing from one level to another is of a predefined precedence, previous levels that have been completed are omitted from the patient profile.
- $\text{Location}_i$: Current location of the player (i.e., $\hbox {Patient}_i$) has to be known to define the precedence of an object and to guide them to achieve the task.

#### **Definition 3**

$\text{Therapist}_j$ $\in$ {Therapist} is a tuple implemented for the {Patient}s’ supervision to include: $\langle$ $\hbox {ID}_j$, $\hbox {Experience}_j$, $\hbox {Involvement}_j$, {Patient}, {Level} $\rangle$

- $\text{ID}_j$: The therapist has to have his/her own profile that is different from other therapists or psychological centers. This will give the therapist an access to the patient profiles and progress reports to allow for further evaluations.
- $\text{Experience}_j$: Experience level of the therapist is useful in allowing access to more complex/detailed data of the patients.
- $\text{Involvement}_j$: The level of engagement within the treatment process which allows the therapist to get access to the reporting progress along the way of the patient assigned treatment.
- ${\{\text{Patient}\}}_j$: The set of patients that $\hbox {Therapist}_j$ is allowed to supervision. Those are $\subseteq$ {Patient} involved with the treatment and currently assigned within the environment.
- ${\{\text{Level}\} }_j$: The therapist is able to assign levels to certain patients as well as to predefine its suitability for the treated patient (e.g., $\hbox {Therapist}_j$ is able to select specified preferable shapes and less saturated ones for a $\hbox {patient}_i$ before starting the session.) This set is assigned directly to a registered patient and does not intervene with original specifications of any level.

#### **Definition 4**

${\{\text{Game-Session}}\}_i$ are a set of treatment sessions a $\text {patient}_i$ goes through within an AR environment to complete a predefined treatment plan from the therapist. It is presented as a tuple of: $\langle$ $\text {Patient}_i$, Type, {Level}, Completion-$\text {Time}_i$, $\text {Tries}_i$ $\rangle$

- ${\text{Patient}}_i$: A player in the treatment which is $\in$ {Patient}. The complete profile is retrieved to include:
  - the current location of the player to define the precedence of an object and to guide them to achieving the task,
  - the set of predefined preferences for a patient considering other psychological disorders that may affect current design and methodology of the treatment plan, and
  - to update the current value of performance the patient has achieved throughout the game.
- Type: The type of the game to be played that has to be suitable for the patient. E.g., drag-and-drop and multiple choices.
- ${\{\text{Level}\} }$: The set of complexity gradients the patient will go through to complete a session starting from the current complexity level defined in the patient profile (i.e., ${\text {Level}}_s$ which is the current level to where the patient has arrived in the treatment plan.)
- Completion-Time: is the time a patient has successfully completed a session which to be used later in measuring the performance index (i.e., to count the response time for ${\text {patient}}_i$.)
- Tries: The number of tries within one session to include correct, incorrect and uncompleted tries. E.g., the number of collected target objects the patient has correctly collected in one session.

#### **Definition 5**

${\{\text{Level}\} }$ is a set of gradient complexities that maximize patients direct attention with each level. It can be seen as a tuple: $\langle$ {Patient}, {Object}, Max-Time, Effects $\rangle$

- $\text{Patient}_i$: A player in the treatment which is $\in$ {Patient}. This allows for assigning a predefined complexity levels based on their current preferences and therapist’s recommendations.
- {Object}: Maximum set of objects used in this level.
- Max-Time: A predefined maximum time for the whole level to be completed or aborted otherwise.
- Effects: Simple directional voice or instructions used for guidance in case of a remote following.

#### **Definition 6**

{Object} is a set of virtual things a player interact with during a session. Specification of those objects is different from one complexity level to another as well as from one type of a game to another. In short, it can be represented as a tuple: $\langle Feature, \{Precedence\}, \{Inheritance\}, Localization \rangle$

- Feature: The structure, size, color, etc. of an object have to be predefined beforehand the start of a session.
- {Precedence}: This sorts the order of the object and which one is visible before another. This will depend on the location and closeness from the player focal point.
- {Inheritance}: The relation between objects in case one object is consecutive after another one. This allows for the visibility of an object after another.
- Localization: The initial distribution of simulated objects around the real environment.

### Process of the reference model

To this point, the components necessary in game design through a reference model have been described. In order for a game utilizing this model to function properly, these components are connected to each other through multiple functions. Here, we elaborate more on the processes of the formal model to assist in the design of the serious treatment games. Before diving into detail, let us restate that repeated pattern of actions performed by one person shapes subsequent resulting behavior. A player's behavior, at this stage, has benefited from role enforcement through prescriptive and proscriptive norms. Even though in a simple form of games, roles still exist. People will be possessing normative behaviors from day-to-day activities and societal boundaries. A game exists, in this sense, to direct the enforced behaviors toward a certain goal that might not directly benefit one player in particular. For this, the formation of a game from governed players behaviors allows them to inherit some regulations. Those inherited regulations, nevertheless, will not directly force players to form certain connections or perform expected actions but will be able to alter behaviors for beneficent new trajectory.

**Fig. 4:**
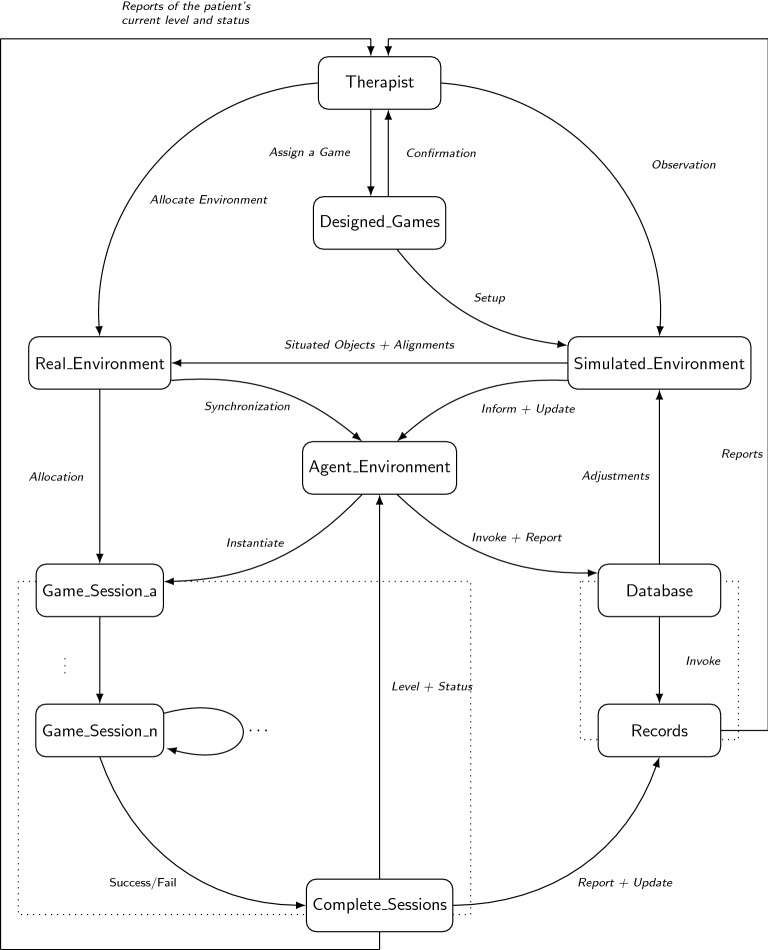
A state-machine diagram to reflect the processes of the profiles that defines the model

The environment profile allows the player to be concerned with the main goal. It is merged with the functioning of a game and does not belong to particular player, which allows for instantaneous updates in the functioning of the game to cope with outside or inside changes (i.e., those changes may result in a transformation of the way a player tackles problems). The environment of a game is in control of generating subsequent tasks that satisfy the goal. Such generations will also consider external needs in order to make sure that a game amalgamates perfectly within the real-life setup and consider internal needs for continual updates in strategies and processes of problems allocations and handling. A goal formation in a game can be a result of two things: (1) a bottom-up approach where a player in the game decides on objectives and works towards achieving those objectives in a suitable environment, or (2) directly or indirectly enforced goal through inheritance of dominant designs. In both cases, game designs result in multiple effects on players’ behaviors that have been identified as regulated protocols through their games. Players may not always commit to such regulations where a huge number of mis-commitments may lead to a transformation of a game to other designs. For that, the goal of a player should be a concrete subset of the game objective. Regulations inherited from design are not static, and a game should allow the environment to keep up with continual changes in the surroundings. In Fig. 4, the therapist generates a set of goals that partly satisfies the observation from the real environment as well as a continual update of this set considering new generated goals. The process of a game session describes how the game functions, and it is an important parameter for its continuation.

By the time a plan is set up for a given goal and is ready for execution, the process of allocation will choose the most fitted patients for each task considering their given profiles as well as the profiles of all tasks they aim to perform. The process allows a proper instantiation of game direction when connecting its processes to players. Algorithm 1 states simplified steps of task allocation for each player based on the patient fitness with what is required to complete a certain task. Patients’ profiles retrieved from the database are only for those who are participating in the level, which trims them as $N \subseteq {\mathcal {N}}$. Each player’s fitness for a given game session is determined through their skills as well as autonomy that are given in their profiles. The consideration is in the instantaneous improvements of patients’ skills when performing multiple actions; for this, they are retrieved as initial values. In other words, any improvement of players skills while performing a game session is considered to improve skills at that stage; therefore, therapists are able to update patients profiles accordingly when the level is completed.

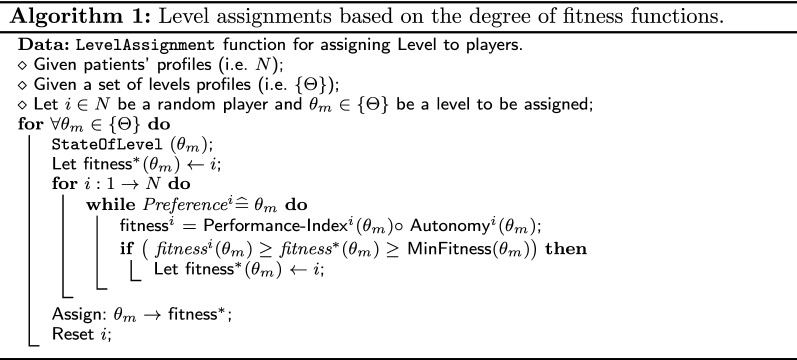



When a plan is determined and levels need to be assigned, the game session should account for the different types of games it produced as well as the expected quality of achieving them. Different types of games have different priority levels and independences that, in result, distinguish the time consumed for processing them. In Algorithm 1, the function—$\mathtt {StateOfLevel}$ is for levels comparison that covers the given object’s Precedence and Inheritance presented in their profiles and sorts them for assignment. The presented autonomous determinism of levels is at a stage of the game at start or when transitioning from one level to another, therapists can be with a continual updates of the input parameters throughout the process when needed. After the process of allocating levels, assigned patients may face difficulties processing those levels efficiently. The therapist, at this stage, is responsible for monitoring their activities. There will be a possible reassignment of levels with a low performance measure to patients that might be of a lower fitness levels. The performance measure is case specific, and a single equation that determines it will not be proposed in this work, readers are directed to [54–57] for more details. For this, performances will be described at different levels in a game assuming that the game session will be able to measure them in every stage it goes through [51].

Patients with allocated game sessions will be announcing the current status of their assignments. When a problem occurs after the proposition of the time constraints setup, players are triggering reports of current status of the problem in hand. This function allows therapist to assign different plans that best fit for their patients. Every time a player fails to achieve a level, it will be set in a queue of prioritize levels. A level with higher priority will be assigned first, and repetition may also be applicable. The therapist may allow assignment of a lower priority level when the process of allocating levels with higher priority fails to be completed with multiple reassignment with different plans. In a case where the time allowed for a level completion is taking longer than expected, the therapist will keep track of such scenarios to better identifying the problem. Some levels might be put on hold for directing attention to another with higher priority.

As the patient engage in a non-cooperative game with the system, such parameters illustrated in Fig. 1 may occur resulting in the patient failing to choose one strategy over another as no dominant strategy exists. Since it does not exists a single move as the best strategy for either player, mixed strategy is used to reflect the probability distribution over all possible moves. The utility of the patient ($U_{patient}$) in a case of revealing their strategy first is in the range between $-3$ and 2: $-3 \le U_{patient} \le +2$. Instead, the probability distribution is used for the two player: $p \mapsto Patient$ and $q \mapsto Agent$. The concern now is the value that the patient should choose for *p* because it will have infinite number of choices for any value of *p* as it cannot play a strategy that allows the opponent to minimize and that is only possible if both terminals are equal which will confuse the agent and does not allow it to take advantages. Since a player/agent has two strategies to choose from a player/agent considers the probability *p* and *q* for the $\Box$ shape strategy and the opposite $1-p$ and $1-q$ for the $\bigcirc$ shape strategy. We will then result with the following:

$$\begin{aligned} 
\begin{aligned} 
U_{patient}(\Box,(p,1-p)) &= U_{patient}(\bigcirc, (p,1-p)) \\
2p - 3(1-p)&= -3 p + 4 (1-p) \\ 
p&= \frac{7}{12} \implies 1 - p = \frac{5}{12},\\
U_{agent}(\Box,(q,1-q)) &= U_{agent}(\bigcirc, (q,1-q)) \\
2 q - 3 (1-q)&= -3 q + 4 (1-q) \\
q&= \frac{7}{12} \implies 1 - q = \frac{5}{12}. 
\end{aligned} 
\end{aligned}$$

The resultant values are then given as feedback to the probability matrix of the game. The following illustrates the process.

$$\begin{aligned} \begin{aligned} U_{patient}&= \left[ \left( 2 \times \frac{7}{12} \times \frac{7}{12}\right) + \left( -3 \times \frac{7}{12} \times \frac{5}{12}\right) \right]\\ &\quad + \left[ \left( -3 \times \frac{5}{12} \times \frac{7}{12}\right) + \left( 4 \times \frac{5}{12} \times \frac{5}{12}\right) \right] \\&= - \frac{1}{6}. \end{aligned} \end{aligned}$$

The expected value is the utility of the game in the range of $-\frac{1}{6} \le U_{patient} \le -\frac{1}{6}$. Therefore, the utility of the patient $= - \frac{1}{6}$. The irrationality and instability in patient’s actions are best modeled through mixed strategies as randomness and secrecy that are naturally occur.

The stage of session completion will compare the current value of a session’s performance with the optimal performance showing in the level profile, and report it to the therapist. The status of completion or failure to complete the level triggers a report of the current status to the agent which will also calculate the player performance and compare it with the minimum performance expected from the patient. When the current performance of a patient exceeds the threshold of the minimum performance or higher, this patient is considered to be performing productively, i.e., the improvement in the patient performance will improve its productivity. Low productivity level is an important internal effect when updated by the agent in the patient’s record will allow the therapist to adopt or plastically transform an environment through different patterns for it to perform better. Plasticity is a group of processes that change the game structurally in to maintain acceptable performances, which in result will require an update to all the profiles in the reference model. The change of performance can be observed at different levels. Predominant forms that affect it most are the result of patients’ distractions. At the patient level, the agent will continually monitor session performances and as needed reassign sessions to each player. In part, a patient’s performance is determined by how well the level is achieved. Reassignments will attempt to augment session achievement; that is, positive session completion will increase level performance.

## Summary and future direction

State-of-art treatment methodologies for many psychological disorders that involve attention primarily generate abroad assessment knowledge as a reflection of the overall effectiveness which makes it challenging to determine the best treatment method. The deployment of instantaneous treatment method provides extra practical implications unforeseen in traditional ones. Although the superiority claim of that method among alternatives is not fully substantiated in this article, a reference model was introduced, and processes of that model were extensively discussed. The design of AR-based treatments with tools that are customized for different patients’ severity level is supported by current evidence on the age specificity of some treatment effects. Hence, therapists are now able to navigate among them and choose the best fit and more suitable for their patients without loosing on effectiveness. Apart from the continuous monitoring as an integral part of an effective treatment approach, positive outcomes of a small or perhaps not existing difference in effectiveness between traditional and AR treatment are that (a) therapists will not have to restrict the patients to a specific treatment method allowing for more options to choose from to find the best fit that is suitable for a specific patient needs, and (b) it allows for more flexibility in adopting to treatment environments and availabilities without affecting the success of the treatment process. Experimental studies that systematically investigate the effectiveness and accessibility of treatment approaches should reduce methodological bias through the moderation of effects resulted from key design features, e.g., randomizations and standardized testing.

In conclusion, the manuscript presented and examined an augmented reality serious game approach as a cognitive behavioral therapy for patients with ADHD. The topic of focus is intriguing and timely, as immersive reality approaches to the treatment of ADHD, as well as other psychological conditions, present promise for improving the reach and impact of mental health treatments, particularly interventions based on evidence-based psychological approaches like CBT. Thus, the paper proposed a theoretical cognitive model that supports the enhancement of patients’ behaviors of whom they have a predefined ADHD with the utilization of a game-based augmented reality environment. The purpose is to support traditional CBT with a more advanced virtual one to accelerate recovery time and to provide a more cost-efficient solution for patients and sustainable health economy to support agencies. The architecture will prompt designs that achieve an excellent accessibility level to every patient in need as they mimic the therapist roles by applying advancements of intelligent virtual agents as well as immersive reality techniques that provide it with features: adaptiveness, smartness, responsiveness, and accuracy. Other advantages are availability and assurance of the therapist’s level-of-experience which cannot be guaranteed in traditional CBT. The consideration in the near future is to apply this model to articulate immersive reality serious games for applied—ethically approved—experimentations with ADHD patients which will support this area with valuable data and interesting findings.

## Abbreviations

ADD: Attention deficit disorder
ADHD: Attention deficit hyperactivity disorder
ASD: Autism spectrum disorder
CBT: Cognitive behavioral therapy
VR: Virtual reality
AR: Augmented reality
ABM: Agent-based model

## Footnotes

1 It tests mixed reality apps on your PC without a physical HoloLens: https://docs.microsoft.com/en-us/windows/mixed-reality/using-the-hololens-emulator.

2 A cross-platform game engine: https://unity3d.com/.
